# Foxp4 Is Dispensable for T Cell Development, but Required for Robust Recall Responses

**DOI:** 10.1371/journal.pone.0042273

**Published:** 2012-08-13

**Authors:** Karla R. Wiehagen, Evann Corbo-Rodgers, Shanru Li, Elizabeth S. Staub, Christopher A. Hunter, Edward E. Morrisey, Jonathan S. Maltzman

**Affiliations:** 1 Department of Medicine, Perelman School of Medicine, University of Pennsylvania, Philadelphia, Pennsylvania, United States of America; 2 Department of Pathobiology, School of Veterinary Medicine, University of Pennsylvania, Philadelphia, Pennsylvania, United States of America; Johns Hopkins University School of Medicine, United States of America

## Abstract

Transcription factors regulate T cell fates at every stage of development and differentiation. Members of the Foxp family of forkhead transcription factors are essential for normal T lineage development; Foxp3 is required for T regulatory cell generation and function, and Foxp1 is necessary for generation and maintenance of naïve T cells. Foxp4, an additional member of the Foxp family, is highly homologous to Foxp1 and has been shown to dimerize with other Foxp proteins. We report the initial characterization of Foxp4 in T lymphocytes. Foxp4 is expressed in both thymocytes and peripheral CD4^+^ and CD8^+^ T cells. We used a CD4Cre mediated approach to evaluate the cell autonomous role for Foxp4 in murine T lymphocytes. T cell development, peripheral cellularity and cell surface phenotype are normal in the absence of Foxp4. Furthermore, Foxp3^+^ T regulatory cells develop normally in Foxp4 deficient animals and naïve Foxp4 deficient CD4 T cells can differentiate to inducible T regulatory cells *in vitro*. In wild-type T cells, expression of Foxp4 increases following activation, but deletion of Foxp4 does not affect T cell proliferative responses or *in vitro* effector T cell differentiation. *In vivo*, despite effective control of *Toxoplasma gondii* and acute lymphocytic choriomeningitis virus infections, effector cytokine production during antigen specific recall responses are reduced in the absence of Foxp4. We conclude that Foxp4 is dispensable for T cell development, but necessary for normal T cell cytokine recall responses to antigen following pathogenic infection.

## Introduction

The Foxp family of forkhead transcription factors regulates diverse cell fate decisions [1], [2], [3]. The four Foxp family members are characterized by a conserved leucine zipper and zinc finger domain, as well as the highly conserved forkhead DNA binding domain [4]. In the immune system, members of the Foxp family regulate gene programs important for normal development and function. Several groups have demonstrated the importance of Foxp3 for the development and function of CD4^+^ T regulatory cells [5], [6], [7]. Foxp1 has essential roles in both B and T lymphocyte development and acts as a transcriptional repressor of the *il7r* gene, which encodes the alpha chain of the interleukin-7 receptor [8], [9]. Furthermore, T lymphocyte-specific deletion of Foxp1 in naïve mice results in an activated phenotype, including upregulation of the activation marker CD44, acquisition of effector functions, and increased apoptosis. Related Foxp member, Foxp4, regulates developmental programs in heart and lung tissues, its deletion resulting in early embryonic lethality [10]. Foxp4 is also expressed in lymphoid tissues, and shares a high degree of homology with Foxp1 [4]. However, the function and role of Foxp4 in T cells has not been described.

Foxp proteins bind DNA as dimers and generally act as transcriptional repressors. Dimerization occurs via the conserved leucine zipper domain [11]. Ectopic overexpression studies have shown that Foxp3 can form heterodimers with Foxp1, as well as homodimers, by means of leucine zipper interactions [12]. Similarly, Foxp4 forms homo- or heterodimers with Foxp1 and Foxp2 in non-lymphoid cells [10]. Foxp family members regulate gene expression by participating in multiprotein complexes. Multiple domains, including the conserved Foxp forkhead domain, mediate interactions between Foxp3 and a variety of partners: NFAT, NFκB, Runx family members, retinoic acid-related orphan receptor (ROR), leading to functional alterations in gene transcription [13], [14], [15], [16], [17]. Direct interaction between Foxp4 and histone deacetylases (HDAC) has also been reported in transfected fibroblasts, but not in lymphocytes [11].

Here we provide the first description of Foxp4 expression and function in T lymphocytes. We show that Foxp4 is expressed in immature thymocytes and in mature T lymphocyte subsets. In contrast to studies of previously reported Foxp family members, Foxp4 deficient thymocytes undergo normal development to become naïve, quiescent peripheral T cells, which demonstrate normal proliferation and upregulation of activation markers upon TCR stimulation. Foxp4 expression has no effect on development of Foxp3^+^ T regulatory cells, induction of Foxp3 in naïve CD4 T cells *in vitro*, or Treg function. Loss of Foxp4 does not immediately expose functional disruption of partner transcription factors. Mice with Foxp4 deficient T cells control infection with *T. gondii* and lymphocytic choriomeningits virus (LCMV). However, the capacity to produce effector cytokines is reduced in peripheral T cells generated by either pathogen and those found in the CNS of *T. gondii* infected animals.

## Materials and Methods

### Mice

Foxp4^FLOX^ mice [18] were generated by introducing loxP sites flanking exons 12,13 encoding forkhead DNA binding domain by standard recombineering [19]. A 5′ loxP site was introduced in the intron between exons 11 and 12, and a 3′ loxP site was introduced in the intron between exons 13 and 14. Southern blot analysis with both 5′ and 3′ probes were used to verify homologous integration. Correctly targeted ES clones were injected into blastocysts to generate chimeric mice, which were further bred to C57BL/6 mice with successful germline transmission of the mutant Foxp4 allele. The neomycin resistance cassette was subsequently removed by intercrossing with Flpe transgenic mice (JAX ACTFLPe-9205Dym/J). CD4-Cre mice were purchased from Taconic [20]. Foxp4^null^ mice have been described [21]. All mice used were backcrossed to C57BL/6 for 1–2 generations. Mice were housed and bred in the University of Pennsylvania mouse facility. All animal experiments were reviewed and approved by the University of Pennsylvania Institutional Animal Care and Use Committee (IACUC protocol number 803447).

### Real-time PCR and probes

Total RNA was extracted from sorted splenocytes or thymocytes derived from adult mice with TRIZOL (Invitrogen, Karlsruhe, Germany) following the manufacturer's instructions. Five nanograms of DNase treated RNA were reverse transcribed in a 20 µl reaction, using First Strand cDNA Synthesis Kit (Amersham Bioscience, Freiburg, Germany). The following TaqMan® assays and primers were employed: Mm00466368_m1 (*FoxP4*), and FAM-MGB 4352933E *(beta-Actin)*. Applied Biosystems Inc. probe sequences are as follows: Foxp4-Fwd (5′ ATCGGCAGCTGACGCTAAATGAGA 3′), Foxp4-Rev (5′ AAACACTTGTGCAGGCTGAGGTTG 3′). Expression levels of Foxp4 were normalized to beta-actin. Fold changes were analyzed with the 2^−ΔΔCt^ method.

### Antibodies and Flow Cytometry

Fluorochrome-conjugated antibodies used for staining were purchased from BioLegend, eBioscience, Invitrogen, BD Biosciences, Beckman Coulter, or Caltag Laboratories. Lymphocytes from spleen were isolated after hypotonic lysis with BioWhittaker ACK solution (Lonza). For tetramer staining, splenocytes were incubated with I-A^b^:gp61 (NIH/Emory Tetramer Facility)at 37°C, 5% CO2 for three hours, washed and stained with APC-CD4, Pacific Blue CD44, PE-Cy7 CD62L and APC-Cy7-CD8 for an additional 30 minutes at 4°C. For intracellular cytokine analysis, cells were stimulated in the presence of Brefeldin A with media alone, 2C11 (BioXCell, West Lebanon, NH), gp61 peptide (Genscript) or with 5 ng/mL phorbol myristate acetate (PMA) and 1 µg/mL Ionomycin. After stimulation, cells were harvested and stained using a Cytofix/Cytoperm kit (Becton Dickinson) according to the manufacturer's instructions. All samples were acquired using an LSRII flow cytometer (Becton Dickinson) and analyzed using FlowJo Version 8 (TreeStar). A cocktail of Pacific Blue-conjugated CD11b (Invitrogen), CD11c (Biolegend), Gr-1 (Biolegend), B220 (Biolegend), NK1.1 (Biolegend) and F4/80 (Caltag) was used to identify lineage negative thymocytes for sorting and real-time PCR.

### Proliferation assay

Spleens were harvested and dissociated into single cell suspension between the frosted sides of two glass slides. Erythrocytes were lysed by hypotonic shock, and splenocytes were washed in T cell media (RPMI [Invitrogen] with 10% FBS, 1% penicillin/streptomycin, 10 mM HEPES, and 1×10^−5^ M 2-ME). Cell counts were performed using a hemocytometer and Trypan Blue dead cell exclusion. Cells were labeled with CFSE by washing with room temperature PBS, resuspending at 1×10^7^ cells/ml PBS, and mixing with an equal volume of PBS containing CFSE (2 µM final concentration). Cells were gently mixed continuously for 3 minutes. The reaction was quenched with 100% FBS, and cells were gently mixed for an additional 2 min in the presence of quench before centrifugation. CFSE-labeled unfractionated splenocytes were plated at 1×10^6^ cells/well in 100 µl T cell media into round-bottom wells of a 96-well plate coated overnight with serial dilutions of anti-CD3 (2C11) in PBS and washed three times with PBS. On the fourth day, cells were harvested, stained, and analyzed by flow cytometry. Live cells were detected by flow cytometry by staining with LIVE/DEAD Fixable Aqua Dead Cell Stain Kit (Invitrogen).

### Generation of iTreg

Single cell suspensions were prepared from spleens as described above, and cells were stained with FITC-CD4 (eBioscience), PerCPCy5.5-CD25 (BD Pharmingen), Pacific Blue-CD44 (Biolegend), and APC- or PECy7-CD62L (BD Pharmingen or Biolegend, respectively). Naïve T conventional cells, defined as CD4^+^CD25^−^CD44^lo^CD62L^hi^, were separated using flow cytometric sorting on Aria (BD Biosciences), and plated in a 48-well flat bottom plate. Cells were plated at 0.5×10^5^ cells/well in a total of 400 µL T cell media (RPMI [Invitrogen] with 10% FBS, 1% penicillin/streptomycin, 10 mM HEPES, and 1×10^−5^ M 2-ME) including stimulus. Wells were pre-coated with 5 µg/ml anti-CD3 (2C11) and cultured in the presence of 5 µg/mL soluble anti-CD28, 50 U/mL IL-2 (R&D) in the presence or absence of 5 ng/mL TGFβ (R&D). After 4 days, wells were harvested and cells were stained for cell surface expression of CD4 and CD25, and intracellular Foxp3 protein using Invitrogen anti-Mouse/Rat Foxp3 Staining Set (APC) as per manufacturer's instructions. Live cells were detected by flow cytometry by staining with LIVE/DEAD Fixable Aqua Dead Cell Stain Kit (Invitrogen).

### In vitro T cell activation marker upregulation

Whole splenocytes were isolated, counted, and suspended at 1×10^7^ cells/mL. Wells of a 96-well round bottom plate were coated overnight with 1 µg/mL anti-CD3 (2C11) and washed three times with PBS before plating. 1×10^6^ unfractionated splenocytes in 100 µL T cell media were plated on coated wells with 5 µg/mL soluble anti-CD28, or left unstimulated in media in uncoated wells. After a 24-hour stimulation, half of the wells were harvested and stained for PacificBlue-CD4, APCCy7-CD8, PerCP-Cy5.5-CD69, PECy7-CD127, FITC-CD44, APC-CD62L for polychromatic flow cytometry on LSRII. Four days after plating, the remaining wells were harvested and cells stained with the same cocktail.

### Infection

*T. gondii*: An i.p. injection of 20 cysts of *T. gondii* ME49 strain, isolated from brains of infected CBA/CaJ mice, was used for infection. For measurement of parasite burden, the 35-fold repetitive *T. gondii B1* gene was amplified by real-time PCR as described [22]. Soluble tachyzoite antigen was prepared from tachyzoites of the RH strain as described [23]. BMNCs from individual chronically infected mice were isolated in accordance with a published protocol [24].

LCMV: Eight – ten week old mice received 2×10^5^ plaque-forming units (PFUs) of LCMV-Armstrong by intraperitoneal injection.

### Analysis of IFNγ production by ELISA

A two-site enzyme-linked immunosorbent assay (ELISA) was employed to assay levels of IFN-γ as previously described [25], [26]. Briefly, spleens were dissociated in complete RPMI (10% heat-inactivated fetal calf serum, 2 mM glutamine, 1,000 U of penicillin per ml, 10 µg of streptomycin per ml, 0.25 mg of amphotericin B [Fungizone], 10 mM HEPES [Gibco, Grand Island, N.Y.], 1 mM sodium pyruvate, 1% [vol/vol] nonessential amino acids [Gibco], 5×10^−5^ M 2-mercaptoethanol) to give a single cell suspension. Brain tissues were processed as previously described [24]. After lysis of erythrocytes, cells were washed twice and plated out at 10^5^/well in a final volume of 200 µl. Cultures were stimulated with anti-CD3 or RH strain Soluble tachyzoite antigen (STAg) or in media for 24 hours. rmIFN-γ was purchased from Genzyme (Cambridge, Mass.)

### Statistical Analyses

Where indicated, *P* values were determined by a 2-tailed unpaired Student *t* test; *P* values <.05 were considered statistically significant. All graphs show averages of the mean ± SD. calculated in Prism software (GraphPad).

## Results

### Foxp4 is expressed in T lymphocytes

**Figure 1:**
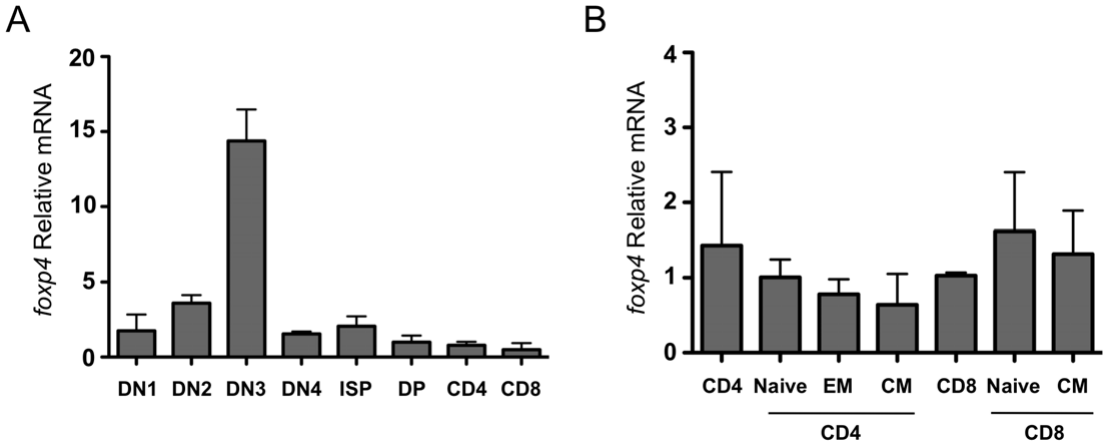
Foxp4 is expressed in lymphoid tissues and T cell subsets. A) Thymocytes from C57BL/6 mice were stained with a lineage cocktail, CD4, CD8, CD44 and CD25, and HSA. Stained cells were collected using flow cytometric cell sorting. Steady state mRNA levels of Foxp4 and ß-actin were determined by TacMan PCR. Foxp4 levels were normalized to β-actin. Double positive thymocytes were given a relative value of 1. All isolated populations were lineage negative. DN1: CD4^−^CD8^−^CD25^−^CD44^+^; DN2: CD4^−^CD8^−^CD25^+^CD44^+^; DN3:CD4^−^CD8^−^CD25^+^CD44^−^; DN4: CD4^−^CD8^−^CD25^−^CD44^−^; ISP: CD4^−^CD8^+^HSA^+^CD5^−^; DP: CD4^+^CD8^+^; CD4: CD4^+^CD8^−^; CD8: CD4^−^CD8^+^HSA^−^CD5^+^. B) C57BL/6 splenocytes were stained with CD4, CD8, CD44, CD62L and CD25 and collected by flow cytometry. Relative levels of Foxp4 were determined as in (A). Total CD8^+^ were given a normalized value of 1. Naive: CD44^lo^CD62L^hi^, EM: CD44^hi^CD62L^lo^; CM: CD44^hi^CD62L^hi^. Error bars represent standard deviation among triplicate wells from TacMan assay. Data is representative of two independent experiments.

We first asked whether Foxp4 is expressed in lymphoid tissues. Protein expression was initially assessed by immunohistochemistry in thymic tissues of fetal mice at age E18.5. Transverse sectioning showed Foxp4 expression in the thymus, particularly in cortical regions (preliminary data not shown). We then examined Foxp4 expression in highly purified T lymphocyte subsets during development in the thymus (Figure 1*A*). Steady-state mRNA levels of Foxp4 were assessed using a TaqMan-based real-time PCR assay. Thymocytes were lineage gated to exclude CD11c, CD11b, or F4/80 expressing cells, and sorted from eight-week-old mice by flow cytometry based on expression of CD4 and CD8. CD4^−^CD8^−^ double negative (DN) thymocytes were further separated based on CD25 and c-Kit expression. Foxp4 mRNA was expressed at detectable levels in all thymocyte populations with the highest levels found in the CD4^−^CD8^−^CD25^+^c-Kit^−^ (DN3) subset. Following β-selection, Foxp4 expression then declines but remains constitutively expressed in single positive T cell subsets.

Previous studies using unfractionated splenocytes have shown that peripheral lymphocytes express Foxp4 [27]. To better understand the expression of Foxp4 in T cell subsets, real-time PCR was used to determine relative Foxp4 levels in various sorted T cell populations. Foxp4 mRNA is expressed in both peripheral mature CD4 and CD8 T cells (Figure 1*B*). Based on CD44 and CD62L expression, these populations can be further divided into subsets of CD44^lo^CD62L^hi^ naïve, CD44^hi^CD62L^lo^ effector memory (EM), and CD44^hi^CD62L^hi^ central memory (CM). While expressed in all sub-populations, no statistically significant differences in Foxp4 levels were detected in either naive CD4 or CD8, EM or CM T cells. Therefore, Foxp4 expression is regulated throughout thymic development decreasing in the most mature cells and is constitutively expressed in unmanipulated mature T cells.

### Foxp4 can be efficiently deleted in thymocytes

**Figure 2:**
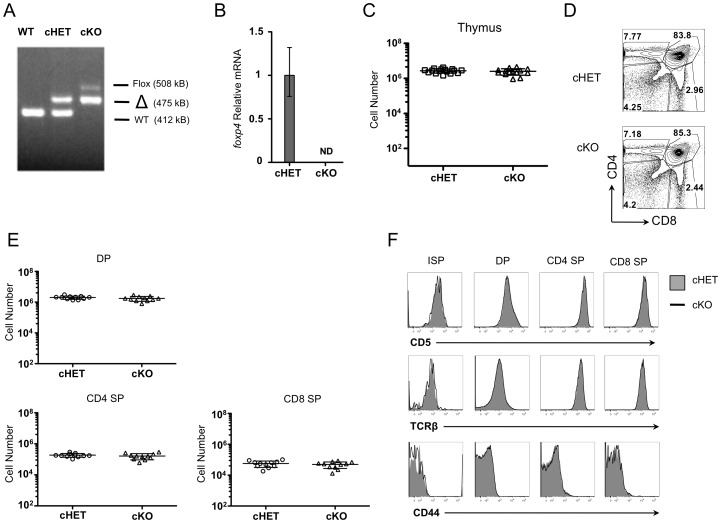
Deletion of Foxp4 at the DP stage does not alter thymocyte development. A) DNA isolated from cHET and cKO thymocytes was amplified using primers to detect wild-type, floxed, and deleted allelic sequences. Band sizes and identities are indicated. Representative of ten experiments. B) RNA isolated from WT (C57BL/6) and cKO thymocytes was assessed for Foxp4 by real time PCR. ND = not detectable. Representative of three experiments. C) Total cellularity was assessed in both cHET and cKO thymi from four-week-old littermates. Each point represents a single mouse. Mean and standard deviation are indicated. D) Thymocytes were stained for CD4 and CD8 and analyzed by polychromatic flow. Contour plots shown are previously gated on live, singlet-gated cells, negative for myeloid lineage markers (CD11b, CD11C, CD19, B220). Gated frequencies are indicated. Representative of twelve cHET and twelve cKO mice. E) Absolute numbers of thymocyte populations were determined. Each point represents an individual mouse. F) Thymocytes were stained with CD4, CD8, CD5 and HSA. Cells are gated on populations as indicated at the top of each column and assessed for CD5, TCRβ, and CD44 expression. Solid histograms are from cHET and bold lines are from cKO mice. Representative of 12 cHET and 12 cKO mice.

To investigate the role of Foxp4 in T lymphocyte development and function, conditional deletion via Cre-loxP was used to generate Foxp4 deficient T cells. Germline deletion of Foxp4 results in embryonic lethality at day E12.5 due to cardiac defects [10] necessitating a conditional approach. To study the effect of Foxp4 deletion specifically in the T lineage, we used mice with loxP sites flanking exons 12–13 which encode the forkhead domain (Figure S1). Foxp4^flox^ mice were intercrossed with CD4Cre mice in which the Cre recombinase is under the transcriptional control of the *cd4* promoter [20]. Under *cd4* promoter regulation, Cre is first expressed at the transition from the DN→double positive (DP) stage in the thymus, leading to gene deletion in DPs, more mature SP thymocytes, and peripheral T cells. To increase likelihood of generating Foxp4 null cells, we used mice carrying one germline null allele and one floxed allele (Foxp4^Flox/null^ CD4Cre^+^, termed cKO). In all experiments, we compared cKO animals to conditional heterozygous (Foxp4^Flox/+^CD4Cre^+^, cHET) littermates, which expressed one wild-type allele and one floxed allele. Following Cre-mediated deletion, cHET T cells express one allele of Foxp4, while cKO mice lack both genomic Foxp4 alleles. To confirm efficient deletion of Foxp4, we first isolated total thymocytes (greater than 95% DP and SP cells) and evaluated deletion by PCR on genomic DNA. Deletion of Foxp4 was verified by detection of a wild-type Foxp4 PCR product at 412 kb, a Foxp4^FLOX^ product including the inserted flanking loxP sites at 508 kb, or the deleted Foxp4^Δ^ product at 475 kb in total thymocytes and purified peripheral CD4 T cells from cKO mice (Figure 2*A* and data not shown). Furthermore, the level of Foxp4 message was undetectable in RNA isolated from Foxp4 cKO purified splenic CD4 T cells, as determined by real-time PCR (Figure 2*B*). Taken together, CD4Cre mediated deletion results in Foxp4 deficient thymocytes and peripheral T cells.

### Foxp4 is not required for T cell development and peripheral homeostasis

To determine if thymocyte development was normal in the absence of Foxp4, we compared the phenotype and cellularity of thymic populations from cHET and cKO. Total thymic cellularity between cHET and cKO mice was comparable (Figure 2*C*). The relative frequency and absolute numbers of DN, DP and SP thymocytes were similar between both cohorts of mice (Figure 2*D–E*). Loss of Foxp4 resulted in no differences in the expression of CD5 or the TCR β-chain between cHET and cKO mice (Figure 2*F*). We also evaluated CD44 levels as Foxp1^CD4Cre^ conditionally deficient T cells acquire a CD44^hi^ phenotype by the single positive stage of thymic development. In Foxp4 cKO mice, however, CD4 SP and CD8 SP cells maintain low levels of CD44 expression. These data suggest that deletion of Foxp4 during transition from DN to DP has no effect on the development of single positive thymocytes.

**Figure 3:**
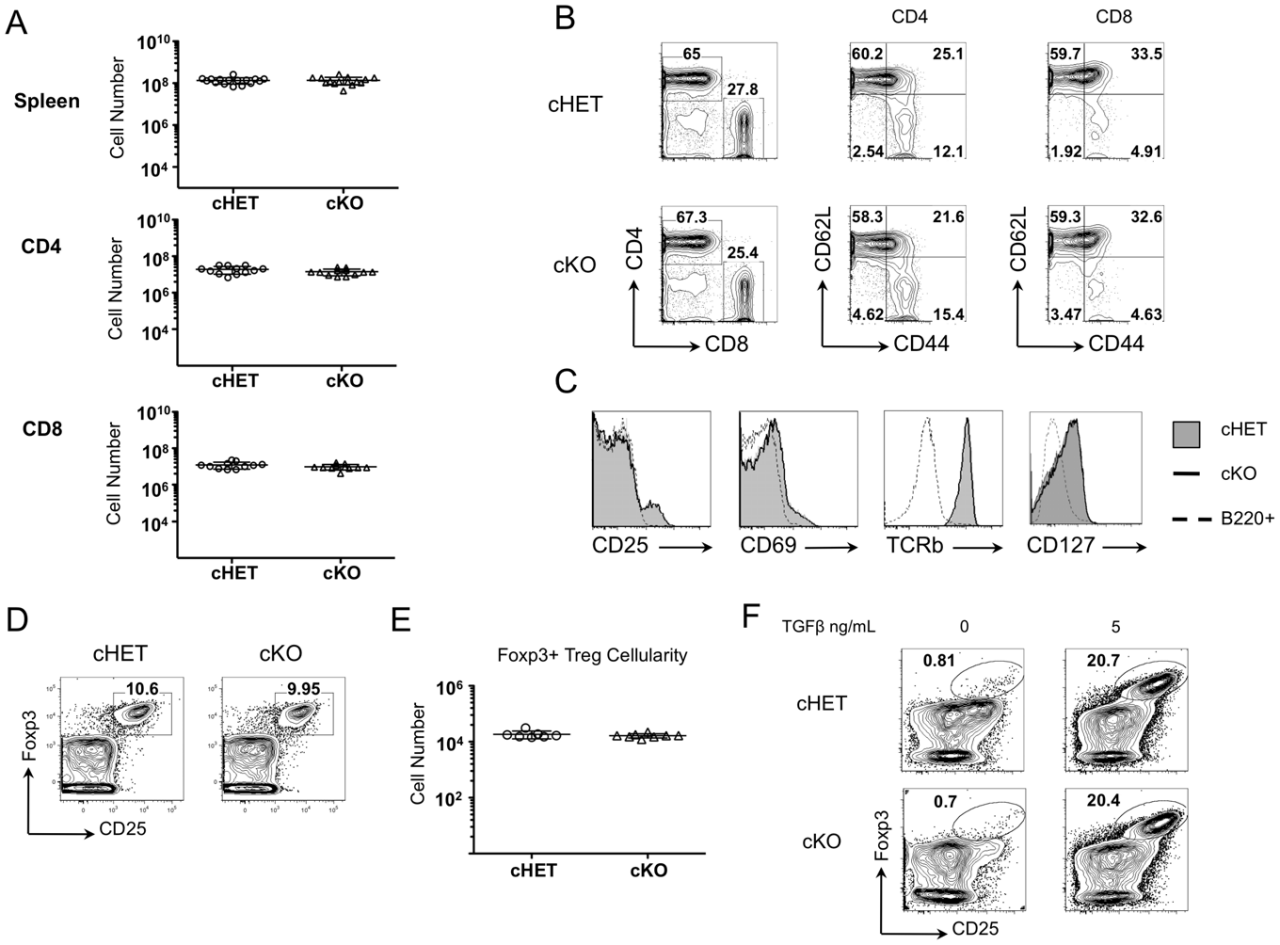
Foxp4 is not required for the generation of peripheral CD4 and CD8 T cells. A) Absolute numbers from spleens from four-week-old cHET and cKO mice, including total spleen cellularity (top panel), numbers of CD4^+^ T cells (middle panel) and CD8+ T cells (bottom panel). B–C) Splenocytes cells from 6–8 week old cHET and cKO mice were stained for expression of CD4, CD8 and CD44 and CD62L or CD25, CD69, TCRβ, and IL-7 receptor (CD127) for immunophenotyping by polychromatic flow cytometry on LSRII. Histograms are gated on live, singlet CD4^+^ T cells. Representative of 4 independent experiments. D) Splenic Foxp3^+^ nTreg cells were identified by flow cytometry based on expression of CD4, CD25 and intracellular staining for Foxp3 protein. Gated frequencies are indicated. Representative of 7 cHET and 7 cKO mice. E) Frequencies of Treg were calculated using the splenic cellularity and relative frequency. Each point represents an individual mouse. Mean and standard deviation are indicated. F) Naïve CD4 T cells from cHET or cKO mice were polarized for four days *in vitro* in the presence of IL-2 with (left) or without (right) TGFβ. Wells were harvested and cells were assessed for expression of CD25 and Foxp3. Plots are gated on live, CD4^+^ cells. Representative of 4 experiments.

We next assessed the effect of Foxp4 deficiency in peripheral T cell populations. Splenic cellularity in cKO mice was similar to that seen in cHETs (Figure 3*A*, top panel). Similarly, the frequency and absolute number of CD4 and CD8 T cells was comparable to controls (Figure 3*A*, middle and bottom panels, Figure 3*B*). In contrast to Foxp1^CD4Cre^ T cells [28], the majority of Foxp4 cKO splenic T cells remain phenotypically naïve, expressing low levels of CD44 and high levels of CD62L. Similar frequencies of CD44^hi^ endogenous memory T cells were present in Foxp4 cHET and cKO mice. Expression of CD25, CD69, and the TCR β-chain on splenic CD4 and CD8 T cells was also similar to controls (Figure 3*C*). Similarly, Foxp4 deletion does not affect levels of the IL-7 receptor on either CD4 or CD8 T cells (Figure 3*C*, right panel). Thus, Foxp4 deficiency does not result in abnormal activation or lead to overt autoimmune pathology, allowing for interpretation of function studies.

Because Foxp family members form homo- and heterodimers with other family members, we next examined whether Foxp4 deficient mice develop Foxp3^+^ regulatory T cells (Treg). The frequency of CD4^+^ T cells expressing the high affinity interleukin-2 receptor (CD25) and Foxp3 was similar between cHET and cKO mice (Figure 3*D*, and data not shown). There was similarly no difference in the total cell number of this population, or in the mean fluorescence intensity of Foxp3 staining (Figure 3*E*, and data not shown). Induced regulatory T cells (iTreg) develop when naïve CD4 cells are activated in the presence of exogenous IL-2 and transforming growth factor β (TGFβ). To assess whether Foxp4 is necessary for the development of iTregs, we cultured naïve CD44^low^ CD4 T cells sorted from cHET or cKO mice *in vitro* with TGFβ and IL-2. After four days under these polarizing conditions, cells were assessed for Foxp3 and CD25 co-expression (Figure 3*F*). Similar frequencies of cHET and cKO CD4 cells acquire the regulatory T cell phenotype after four days. Thus, Foxp4 is not necessary for the development of nTreg or generation of iTreg cells.

### Lack of Foxp4 does not alter *in vitro* cytokine responses or proliferation

**Figure 4:**
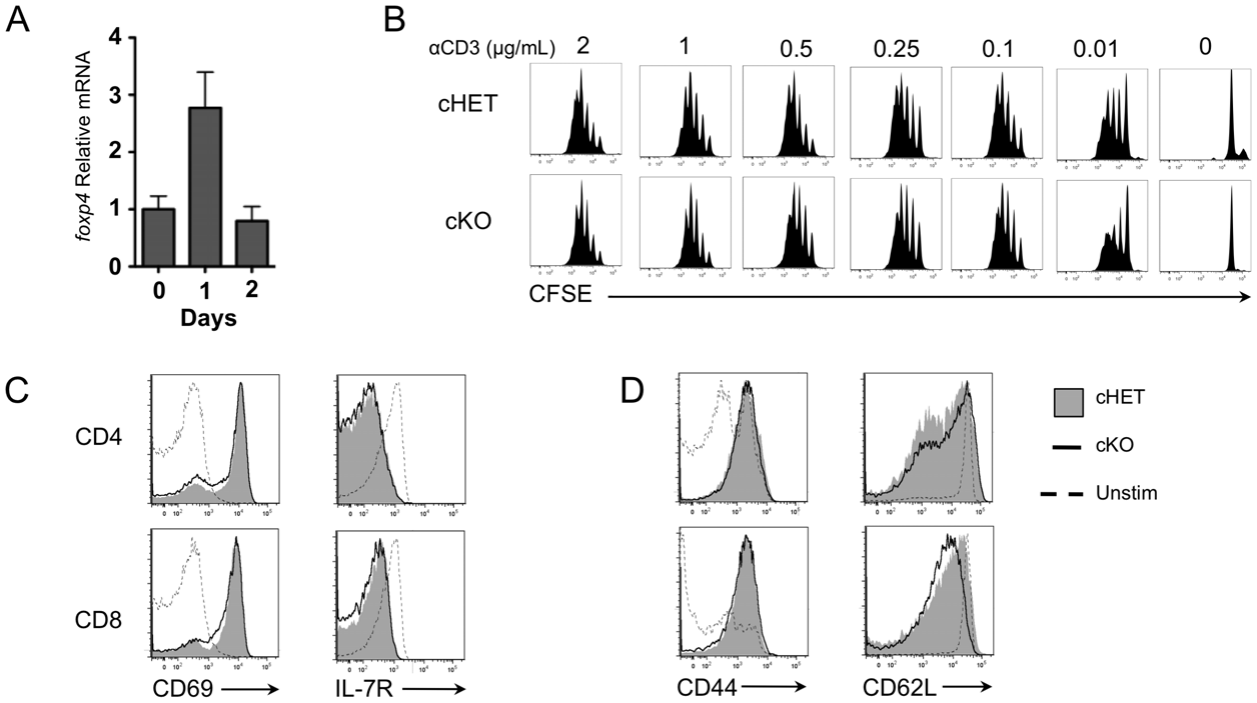
T cell activation induces normal proliferation and effector T cell differentiation in the absence of Foxp4. A) C57BL/6 CD4^+^ T cells were isolated and plated in wells coated with 1 µg/mL anti-CD3 and 5 µg/mL soluble anti-CD28. Wells were harvested on the indicated days. RNA was isolated and cDNA was generated as described in Materials and Methods. Foxp4 relative to β-actin transcript levels were normalized to day 0. Representative of 2 experiments. B) CD4^+^ T cells were isolated from spleens of cHET and cKO mice, labeled with CFSE, and cultured four days in wells coated with indicated concentrations of anti-CD3 and 5 µg/mL soluble anti-CD28. Dilution of CFSE was assessed on day 4. Representative of 4 cHET and cKO mice. C) CD4^+^ T cells from cHET and cKO mice were stimulated overnight in wells coated with anti-CD3 plus soluble anti-CD28. Harvested cells were stained for CD69 and cytokine receptor IL-7R. Histograms are gated on CD4 or CD8 TCRβ^+^ cells. Representative of 2 experiments. D) cHET and cKO CD4^+^ T cells were cultured in wells coated with anti-CD3 plus soluble anti-CD28. After four days, cells were stained for expression of activation markers CD44 and CD62L. Representative of 4 cHET and 5 cKO mice.

We next asked whether Foxp4 levels are regulated in response to activation. CD4^+^ T cells from C57BL/6 mice were stimulated with plate-bound anti-CD3 and anti-CD28 and assessed for Foxp4 RNA levels using real-time PCR. Foxp4 message level is increased three-fold at twenty-four hours after TCR stimulation and returns to pre-activation levels by forty-eight hours (Figure 4*A*). The change in Foxp4 expression in response to TCR plus CD28 costimulation suggests that it may be involved in a transcriptional program of activation, so we asked whether Foxp4 deficient T cells undergo normal activation and proliferation. We assessed T cell proliferation by stimulating CFSE-labeled T cells *in vitro* with anti-CD3 and anti-CD28. Foxp4 cKO CD4 and CD8 T cells dilute CFSE comparably to cHET T cells (Figure 4*B* and data not shown). Proliferation of cKO cells was equivalent to cHET cells regardless of the concentration of anti-CD3 used. Activated cKO T cells were then assessed for regulation of cell surface effector markers typically induced during effector responses. Overnight stimulation of CD4 and CD8 T cells resulted in equivalent induction of CD69 and decreased levels of IL-7R (Figure 4*C*). Similarly, splenocytes stimulated for four days exhibited increased CD44 expression and concomitant downregulation of CD62L expression similar to control cHET cells (Figure 4*D*). Together, these data suggest that the loss of Foxp4 does not alter the proliferative response or the profile of cell surface receptors expressed following T cell activation.

### Foxp4 deficient T cells control chronic infection

**Figure 5:**
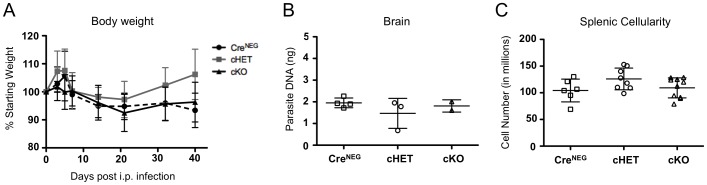
*Toxoplasma gondii* infection does not lead to wasting or lymphopenia in Foxp4 cKO mice. Eight-week-old Foxp4^FLOX/FLOX^Cre^NEG^, cHET and cKO mice were infected with *T. gondii* by intraperitoneal injection of 20 Me49 cysts. A) Weights of mice were assessed longitudinally and plotted relative to the percentage of each respective mouse's starting weight at d0. Mean and s.d. from 9 Cre^NEG^, 12 cHET, and 15 cKO mice. B) Brain tissue was harvested at day 40 post infection and parasite DNA quantitated using SYBR probes specific for *T. gondii*. Levels of *T. gondii* DNA were normalized to a standard curve. Each point represents an individual mouse. Mean and standard deviation are shown. Representative of 3 individual experiments. C) Spleens were harvested at 40 days post infection and total spleen cellularity was determined. Each point represents and individual mouse. Representative of 3 experiments.

We next asked whether T cell specific Foxp4 deficiency alters the physiologic response to pathogenic infection. *Toxoplasma gondii* is an intracellular parasite that elicits a systemic Th1 type immune response but establishes a chronic infection approximately thirty days after infection. To assess the response to chronic toxoplasmosis, *T. gondii* cysts were injected intraperitoneally to cHET and cKO cohorts, and Foxp4^Flox/Flox^ Cre-negative littermates. In the setting of immune dysregulation, toxoplasmosis results in weight loss and increased morbidity. We monitored body weight throughout the course of infection. Weights were then normalized as a percentage relative to starting weight, and averaged across cohorts (Fig. 5*A*). No differences were observed in the weight or morbidity between groups of mice, and no overt immune pathology developed.

To assess parasite clearance, brain tissues were preserved for immunohistopathology and determination of parasite burden. cKO tissues and littermate controls were assessed in a blinded fashion for cyst counts, with no appreciable difference across the cohorts (data not shown). Parasite burden in the brain was assessed by real-time PCR based quantitation of parasite DNA remaining forty days post-infection. Parasite load by DNA amplification indicated similar degrees of chronic toxoplasmosis in cHET and cKO animals (Figure 5*B*). No additional effects on tissue histology or animal survival were observed. These data suggest that T cell specific loss of Foxp4 does not alter the overall ability to control *T. gondii* infection.

To further characterize the immune response, T cells from spleen, lymph node and CNS were analyzed. Overall splenic cellularity, CD4 and CD8 T cell numbers were comparable between cKO and control mice (Figure 5*C* and data not shown). We assessed proliferation by Ki-67 staining of splenic lymphocytes. Similar numbers of Cre^NEG^, cHET and cKO T cells stained positively for Ki-67, suggesting cKO T cell numbers were maintained by normal turnover in response to *T. gondii* (data not shown). CD4 and CD8 T cells from spleens and lymph nodes of Cre^NEG^, cHET and cKO mice showed comparable expression of activation markers CD44 and CD62L (data not shown), further suggesting that Foxp4 is not necessary for the maintenance of peripheral T cells during chronic toxoplasmosis.

**Figure 6:**
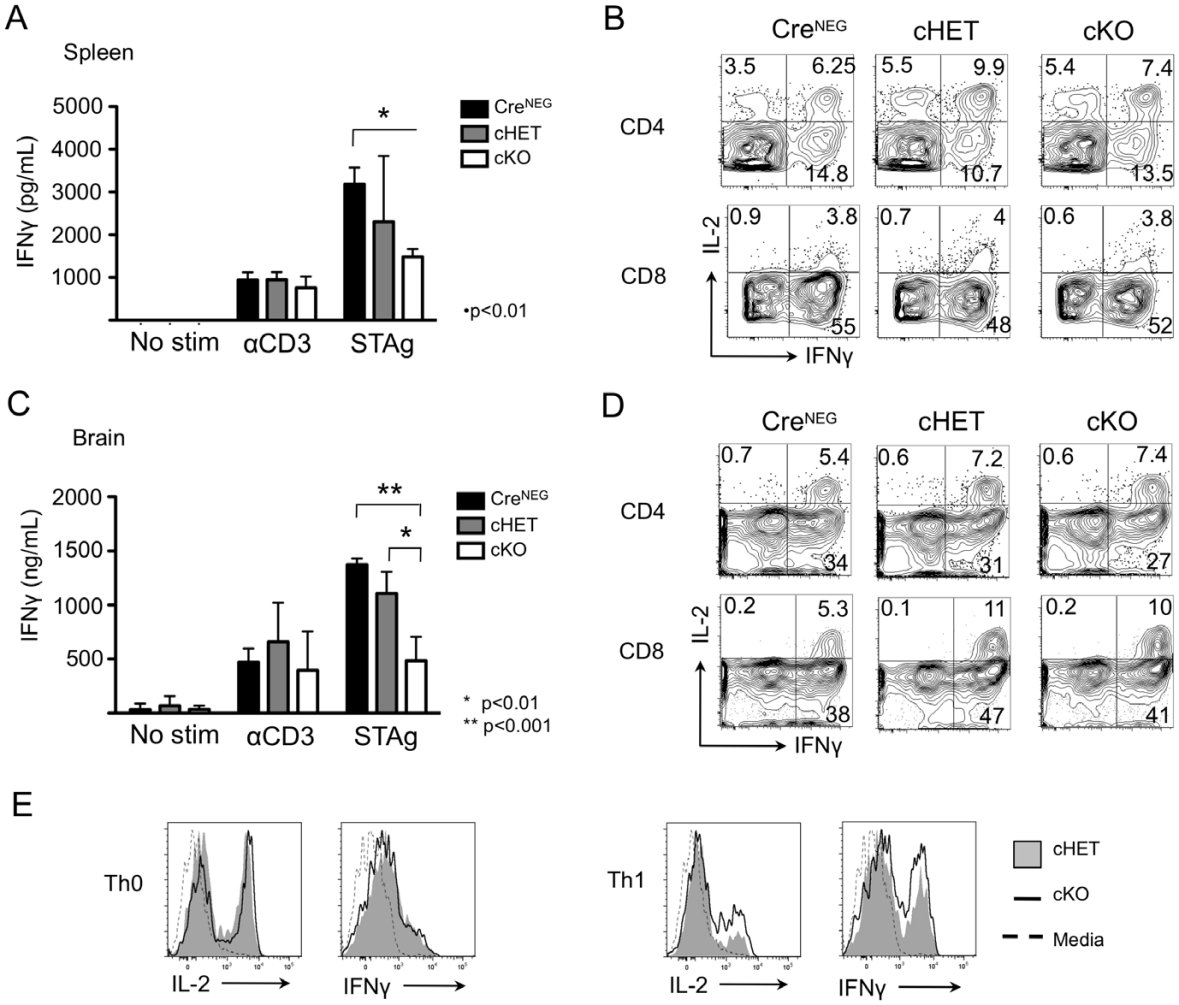
Foxp4 deletion alters recall responses to *Toxoplasma gondii* in the spleen and brain. A and C) Splenocytes and BMNC from mice infected with *T. gondii* 40 days earlier were cultured with anti-CD3, soluble tachyzoite antigen (STAg), or left unstimulated for twenty-four hours. Supernatants were analyzed for levels of IFNγ by ELISA, and normalized to a standard curve. Representative of 3 independent experiments. B and D) Splenocytes and BMNC were stimulated for four hours with PMA and Ionomycin in the presence of Brefeldin A, and assessed for intracellular IL-2 and IFNγ. Contour plots are gated on CD4^+^ or CD8^+^CD3^+^ cells. Relative percentage is shown within each gate. Splenocytes are representative of 9 Cre^NEG^, 12 cHET and 15 cKO. BMNC are representative of 5 Cre^NEG^, 7 cHET, and 7 cKO mice. E) CD8-depleted splenocytes from cHET and cKO mice were cultured in Th0 (left) or Th1 (right) polarizing conditions. Cells were restimulated in the presence of monensin. Histograms are gated on CD4^+^ T cells. cHET (filled histogram); cKO (black solid line); unstimulated (dashed line). Representative of 6 experiments.

We next assessed the differentiation and functionality of Foxp4 cKO effector T cells that had been generated in response to *T. gondii* infection. Cytokine production in cHET and cKO T cells was first assessed by enzyme-linked immunoassay (ELISA). Whole splenocytes were cultured for twenty-four hours in the presence of anti-CD3 or soluble tachyzoite antigen (STAg), or left unstimulated in media (Figure 6*A*). Culture supernatants were assessed for IFNγ, an effector cytokine critical in controlling *T. gondii* infection [29], [30]. STAg stimulation, which is limited to those cells specific for *T. gondii,* resulted in statistically lower levels of soluble IFNγ detectable in cKO versus Cre^NEG^ control. A similar trend toward lower IFNγ was seen when comparing Cre^NEG^ to cHET and cHET to cKO cells, but the difference did not reach statistical significance. Conversely, supernatants from splenocyte cultures stimulated with anti-CD3 contain equivalent levels of IFNγ. These results suggest that Foxp4 may repress the induction of IFNγ in an antigen-specific manner.

The difference seen in IFNγ production could be due to a reduced number of IFNγ producing T cells, a decrease in the per cell production of IFNγ, or due to increased consumption of the cytokine. To determine if cKO T cells had altered cytokine generation on a per cell basis, unfractionated splenocytes were assessed for by intracellular cytokine staining (ICS) for production of IFNγ, IL-2 and tumor necrosis factor alpha (TNFα). Analysis by flow cytometry indicated no difference in the frequency or absolute number of CD4 or CD8 T cells capable of producing the cytokines assayed (Figure 6*B* and data not shown, respectively).

Given that effector function of Foxp4 cKO splenocytes showed a trend toward decreased response to STAg stimulation, we also asked whether the functionality of T cells in the brain, a site at which constant antigen presentation occurs during toxoplasmosis, was altered. We isolated mononuclear cells from the brain (BMNCs) and assessed cytokine production by ELISA and ICS (Figure 6C). Similar to splenic T cells, there was no difference in the levels of IFNγ in response to anti-CD3. In wells stimulated with STAg, IFNγ levels are statistically decreased in cKO BMNCs, relative to BMNC responses from cHET and Cre^NEG^ mice. Foxp4 cKO BMNCs were further assessed by ICS in a four-hour recall response. Cells isolated from the brain tissues generate IFNγ, IL-2, and TNFα at similar frequencies, despite the lower quantity detected by ELISA (Figure 6*D* and data not shown). Therefore, in contrast to total cytokine secreted, the frequency of CD4 and CD8 T cells within BMNC fractions were comparable across cohorts.

During chronic infection with *T. gondii*, Th1 CD4 T cells are an important source of IFNγ, and are required for control of the parasite [31], [32]. Maintenance of IFNγ is necessary to prevent cyst reactivation. Differences in the quality of Th1 effector T cells could account for decreased IFNγ production by cKO cells. To ask whether Foxp4 regulation is important in Th1 differentiation of CD4 T cells, we resorted to an *in vitro* model of T cell polarization. Preliminary experiments demonstrated that CD4 and CD8 cKO T cells were capable of making IFNγ, TNFα and IL-2 immediately *ex vivo* (data not shown). To determine whether Foxp4 deficiency affects CD4 T cell differentiation, CD8-depleted splenocytes from naïve cHET and cKO mice were cultured with cytokine and blocking antibody to polarize cells towards Th0 or Th1 lineages. Cultures were restimulated with PMA and Ionomycin and harvested for intracellular cytokine staining (ICS). T cells stimulated with anti-CD3 and anti-CD28 produced similar levels of cytokine to cHET controls, indicating T cell activation without Foxp4 expression does not result in dysregulated cytokine production (Figure 6*E*, left panels). Both cHET and cKO CD4 T cells cultured under Th1 polarization conditions with IL-12 and anti-IL4 produced IFNγ similarly (Figure 6*E*, right panels). Predicted T helper responses to restimulation suggest Foxp4 is not necessary for differentiation or commitment to T helper lineages. Therefore, the decreased cytokine response by chronically infected Foxp4 cKO cells is not caused by reduced IFNγ production on a per cell basis, deficient Th1 CD4 effector function, or a striking lack of lymphocytes. Rather, Foxp4 deficient cells demonstrated decreased IFNγ responses to STAg stimulation by ELISA regardless of tissue, suggesting a systemic defect in antigen-specific immunity.

**Figure 7:**
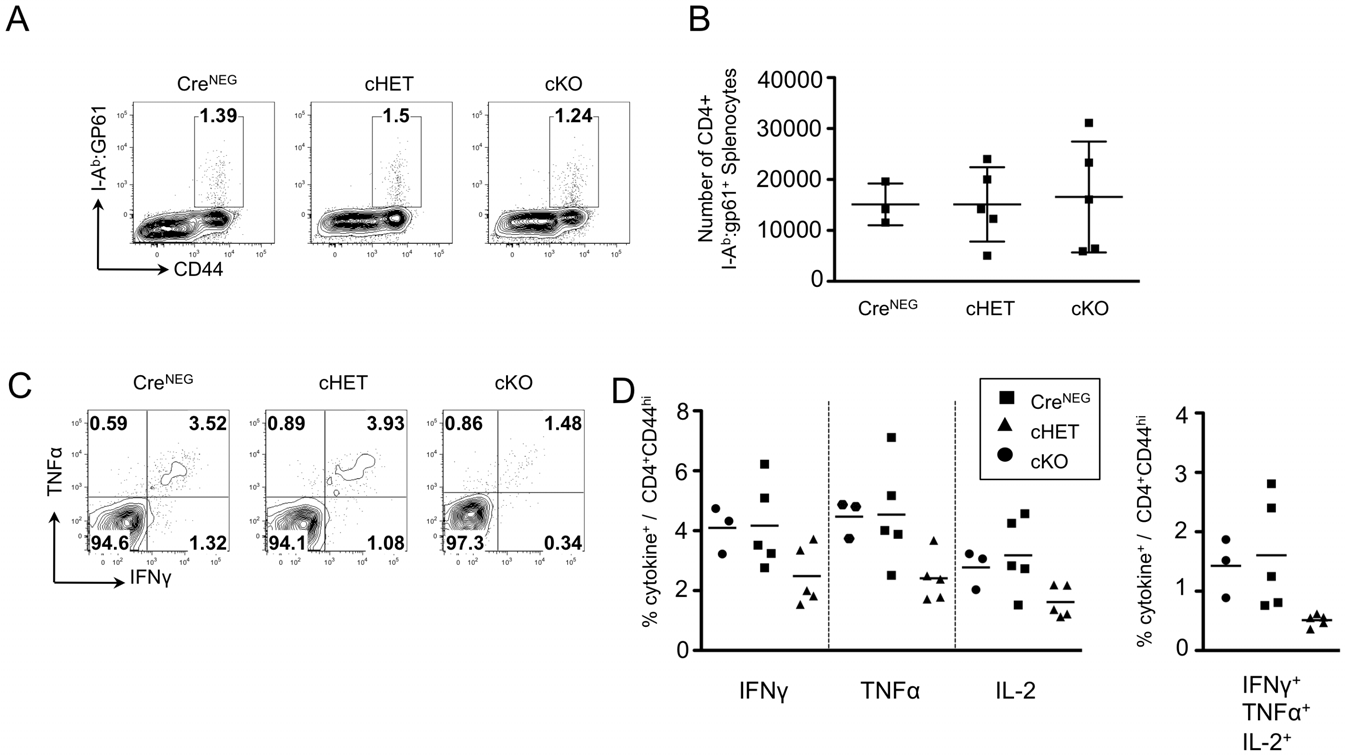
Foxp4 deficient CD4 T cells exhibit reduced memory recall responses following LCMV infection. Foxp4^FLOX/FLOX^Cre^NEG^, cHET and cKO mice were infected with the Armstrong strain of LCMV by intraperitoneal injection at day 0, allowed to clear infection, and to generate memory T cells. At day 30 post-infection spleens were harvested for analysis. A) Frequencies of I-A^b^:gp61^+^ CD44^hi^ CD4 T cells at day 30. Representative of 3 Cre^NEG^, 5 cHET and 5 cKO mice across two independent infections. B) Numbers of I-A^b^:gp61^+^ CD44^hi^ CD4 T cells were calculated. C) Splenocytes harvested at day 30 were restimulated with gp61 peptide for four hours *in vitro*, in the presence of Brefeldin A. Cells were assessed for cell surface expression of CD4, CD8, CD44, and stained intracellularly for IFNγ, TNFα, and IL-2. Representative plots of 3 Cre^NEG^, 5 cHET and 5 cKO across two independent infections. D) Compiled frequencies of cells producing IFNγ, TNFα, or IL-2 (left), or polyfunctional cells producing all three cytokines (right). Cre^NEG^, filled circles; cHET, filled squares; cKO, filled triangles.

We next infected Cre^NEG^, cHET and cKO mice with acute LCMV (Armstrong strain) to determine whether reduced cytokine production in Foxp4 deficient memory T cells is limited to *T. gondii* infection. In contrast to chronic infection with *T. gondii* where pathogen and inflammation persist, LCMV Armstrong is cleared within ten days post-infection. Cohorts were assessed for maintenance and function of memory CD4 T cells. An MHC Class II tetramer∶peptide reagent was used to identify I-A^b^:gp61 specific T cells. At thirty days post infection, comparable frequencies of gp61-specific CD4 T cells were found in Cre^NEG^, cHET and cKO mice (Figure 7A). Total numbers of I-A^b^:gp61 tetramer+ cells were calculated from spleen cellularity and demonstrated similar numbers across all cohorts (Figure 7B). Splenocytes were left unstimulated or stimulated with gp61 peptide and assessed for the ability to generate IFNγ, IL-2 and TNFα alone or in combination (Figure 7C, 7D and data not shown). Despite equal numbers of I:A^b^:gp61 positive cells, fewer cKO T cells rapidly induced effector cytokines relative to either cHET or Cre^NEG^ cells. A trend toward decreased cytokine producing cells was seen for IFNγ, TNFα and IL-2, but it did not reach statistical significance. Frequencies of polyfunctional gp61-specific memory cells were also reduced in the cKO (Figure 7D, right panel). These results suggest that Foxp4 expression is important in memory recall responses following acute or chronic infections.

## Discussion

In this manuscript, we provide the first description of Foxp4 in T lymphocytes. We show that Foxp4 is expressed in T cells and plays a role in effector T cell cytokine responses in a chronic infection. We expected to find Foxp4 cKO T cells with an activated phenotype due to the high degree of homology between Foxp1 and Foxp4 and known heterodimerization in other cell types. Surprisingly, naïve Foxp4 deficient T cells develop normally, indicating Foxp4 is not necessary to maintain T cell quiescence. More recent studies have shown Foxp1 represses IL-7Ra transcription and regulates homeostasis in T cells [33]. Whether repression of IL-7R transcription is due to Foxp1∶Foxp1 homodimers or Foxp1∶Foxp4 heterodimers was not directly addressed here, but our data that deletion of Foxp4 has no effect on either IL-7R levels or on peripheral homeostasis based on steady-state lymphocyte numbers and *in vivo* bromodeoxyuridine labeling (data not shown) suggest that repression of IL-7R transcription and maintenance of a naïve phenotype is Foxp4 independent.

We find that Foxp3 expression in Treg CD4 cells is similarly not affected by Foxp4 deletion. Normal Treg development in Foxp4 cKO mice suggests Foxp3∶Foxp4 heterodimerization is not necessary for the generation or maintenance of natural Tregs *in vivo*, nor in the conversion of CD4 T cells into Foxp3^+^ Tregs *in vitro* by culture with TGFβ and IL-2. In addition, we did not find alterations in the frequency or number of Tregs in chronically infected mice (data not shown), suggesting that Foxp4 deletion does not have an impact on development or maintenance of Tregs under homeostatic or infectious conditions.

Because Foxp4 expression is not necessary for normal generation of peripheral T cells, we were able to evaluate the functionality of Foxp4 deficient T cells using a CD4Cre mediated deletion approach. We expected Foxp4 expression in activated T cells to be important for T cell activation and possibly differentiation given the three-fold increase in expression following stimulation, but Foxp4 deficient effector cells did not appear defective when evaluated by *in vitro* assays. Naïve Foxp4 deficient T cells upregulate activation markers and proliferate normally following TCR crosslinking suggesting that effector T cells can differentiate normally in the absence of Foxp4. Furthermore, CD4 and CD8 T cell cytokine production of IL-2 and IFNγ, respectively, are intact when rechallenged four days after activation. Thus, despite increased expression of Foxp4 with activation, we have found no substantial effect on activation.

To more thoroughly evaluate effector function, we examined Foxp4 deficient T cell responses following pathogenic infection. Following chronic infection with *T. gondii*, Foxp4 cKO mice controlled the infection similarly to controls as evidenced by similar numbers of cysts and quantitatively equal parasite burden. Total cellularity and phenotype of T cells in infected cKO mice were comparable to cHET animals. However, Foxp4 cKO antigen-specific lymphocytes exhibited reduced cytokine responses upon antigen specific rechallenge. While stimulation with anti-CD3 resulted in similar quantities of cytokine detected, STAg stimulation resulted in lower IFNγ responses. On an individual cell basis, Foxp4 cKO CD4 and CD8 T cells were capable of producing cytokine in similar frequencies following short-term PMA and Ionomycin stimulation. This finding is not unique to chronic infection with *T. gondii*. Lower frequencies of Foxp4 deficient memory cells also produced cytokines during recall responses following acute LCMV infection. Furthermore, *in vitro* polarization experiments proved naïve CD4 T helper cells lacking Foxp4 could commit to the Th1 lineage, and maintain IFNγ production upon restimulation. However, the lower quantities of cytokine from twenty-four hour stimulation with STAg suggests Foxp4 deficient cells are less responsive to *T. gondii* antigen.

While the decreased STAg response may suggest Foxp4 is important for the production and secretion of IFNγ by T cells, it does not exclude a role for antigen presenting cells (APCs) or natural killer cells. Expression of CD4Cre generates T lineage-specific deletion of Foxp4, but does not rule out a model in which the ability of CD4 T cells to license APCs is dependent on T cell expression of Foxp4. Mechanisms of CD4 T cell licensing of dendritic cells during chronic infections are not well understood. CD4 T cells boost the ability of APCs to cross-prime and activate cytotoxic CD8 T cells through CD40∶CD40L, and CD80/CD86∶CD28 interactions [34], [35]. In chronic viral infection, CD4 depletion results in loss of long-term CD8 T cell responses, and host animals maintain high viral loads [36]. Similarly, infection of CD4−/− mice with *T. gondii* results in protective acute CD8 T cell responses, but CD4 T cells are required to sustain immunity [37]. In Foxp4 cKO mice, the reduced IFNγ production in response to STAg forty days post-infection may be caused by defects in CD4 T cell help. Interactions with APCs and Foxp4 deficient CD4 T cells may not provide CD4 T cell help signaling to APCs, resulting in suboptimal CD8 T cell priming. If Foxp4 regulates CD4 T cell help, we would expect more impaired CD8 responses at later points post-infection, leading to uncontrolled infection and increased mortality, which was not evident in our shorter infection.

These studies are the first to characterize expression of Foxp4 in T cells and report mildly diminished effector responses in acute and chronically infected Foxp4 deficient mice. Despite previous evidence of Foxp protein dimerization, Foxp1-dependent regulation of naïve T cell maintenance and IL-7R expression is independent of Foxp4 expression. Similarly, induction of Foxp3 expression in CD4 T cells does not require Foxp4. Instead, Foxp4 expression is increased in activated T cells, and *in vivo* studies suggest a role for Foxp4 in the regulation of cytokine responses. While Foxp1 and Foxp3 are both necessary to maintain a dormant immune environment, Foxp4 appears to have a supportive but not primary role in maintaining protective immune responses.

## Supporting Information

Figure S1 **Generation of Foxp4 cHET and Foxp4 cKO mice.** Foxp4 conditional heterozygous (cHET) and conditional knockout (cKO) mice were generated by interbreeding mice carrying Foxp4^FLOX^ alleles with mice expressing the Cre recombinase on the *cd4* promoter, resulting in T lineage specific Foxp4 deletion. Arrows indicate loxP sites flanking exons 12 and 13, which encode the Forkhead DNA binding domain. cHET mice express one wild-type Foxp4 allele and one floxed allele. cKO mice express one floxed allele and are germline null on the second allele. Following Cre mediated recombination, cHET cells retain one copy of wild-type Foxp4 allele whereas cKO T have no functional allele (null and deleted (Δ) alleles).
(TIF)
