# Supplementary figures and images for: Foxp4 Is Dispensable for T Cell Development, but Required for Robust Recall Responses
Source: PLoS One. 2012 Aug 13;7(8):e42273. doi: 10.1371/journal.pone.0042273 (PMC3418275; doi:10.1371/journal.pone.0042273)

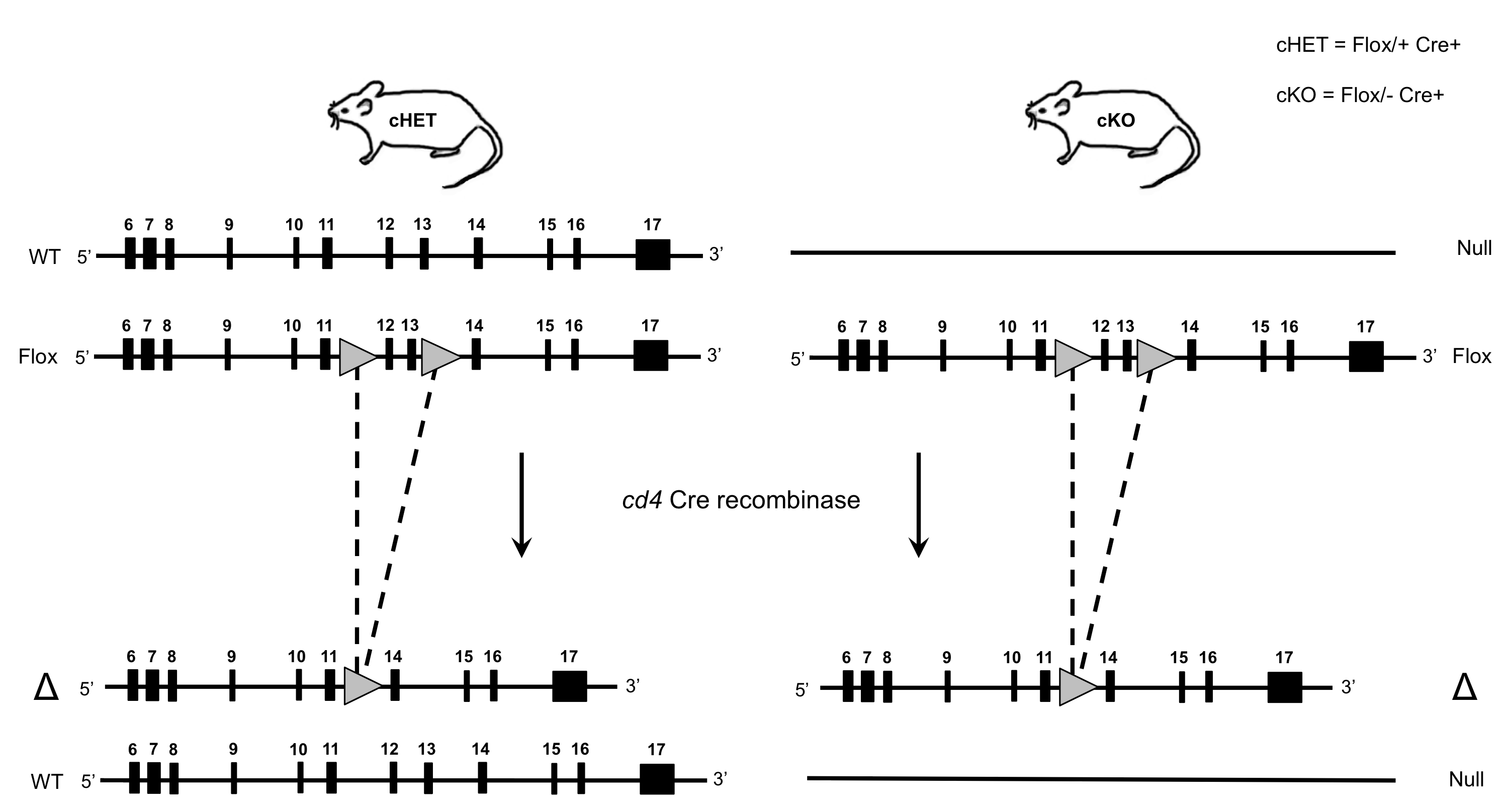

Supplement: Figure S1 — Generation of Foxp4 cHET and Foxp4 cKO mice. Foxp4 conditional heterozygous (cHET) and conditional knockout (cKO) mice were generated by interbreeding mice carrying Foxp4FLOX alleles with mice expressing the Cre recombinase on the cd4 promoter, resulting in T lineage specific Foxp4 deletion. Arrows indicate loxP sites flanking exons 12 and 13, which encode the Forkhead DNA binding domain. cHET mice express one wild-type Foxp4 allele and one floxed allele. cKO mice express one floxed allele and are germline null on the second allele. Following Cre mediated recombination, cHET cells retain one copy of wild-type Foxp4 allele whereas cKO T have no functional allele (null and deleted (Δ) alleles). (TIF) [file pone.0042273.s001.tif]
